# A Systematic Review of Safety-Driven Approaches in Human–Robot Collaborative Systems

**DOI:** 10.3390/s26072079

**Published:** 2026-03-27

**Authors:** Akhtar Khan, Maaz Akhtar, Sheheryar Mohsin Qureshi, Muzzamil Mustafa, Naser A. Alsaleh, Imran Ahmad

**Affiliations:** 1 Department of Electrical Engineering, Iqra Nations University, Peshawar 25000, Pakistan; akhtarkhan1992@gmail.com; 2 Industrial Engineering Department, College of Engineering, Imam Mohammad Ibn Saud Islamic University (IMSIU), Riyadh 11432, Saudi Arabia; naalsaleh@imamu.edu.sa; 3 School of Computing, Engineering and Physical Sciences, University of the West of Scotland, Paisley PA1 2BE, UK; 4 Department of Artificial Intelligence, University of Management and Technology, Lahore 54782, Pakistan; muzzamil.mustafa@umt.edu.pk; 5 Department of Industrial Engineering, University of Engineering and Technology, Peshawar 25000, Pakistan; imranahmad@uetpeshawar.edu.pk

**Keywords:** generative AI, variational auto encoders, large language models, PRISMA, Generative Adversarial Networks

## Abstract

Collaboration between humans and robots (HRC) is advancing rapidly due to the intersection of robotics and generative artificial intelligence (GenAI). The current paper includes a systematic review of 103 studies on the role of generative models, including Generative Adversarial Networks (GANs), Variational Autoencoders (VAEs), diffusion models, and Large Language Models (LLMs) in improving the safety, trust, and adaptability of collaborative robotics using a PRISMA-based systematic approach. The review recognizes four major themed areas of GenAI-based safety frameworks—namely, data-driven simulation to synthesize hazards, predictive reasoning to forecast human motion, adaptive control to reduce risks dynamically, and trust-aware cognition to explain human–robot interaction. Findings indicate that generative models transform robotic safety from a reactive mechanism to proactive, contextual and interpretable systems. Nevertheless, real-time performance, interpretability, standard benchmarking, and ethical assurance are still some of the challenges to be overcome. The paper proposes a taxonomy linking generative modeling layers and physical, cognitive and ethical aspects of HRC safety, and gives a roadmap of certifiable hybrid systems with generative foresight and deterministic control. This synthesis provides a foundation for developing transparent, adaptive, and trustworthy collaborative robotic systems.

## 1. Introduction

Human–robot collaboration (HRC) is one of the paradigms of Industry 4.0 and Industry 5.0, as intelligent robots and people share workspaces and goals, as well as tasks to attain increased productivity, flexibility, and performance. In the last 10 years, sensorimotor control and artificial intelligence technologies have increased the pace of implementation of collaborative robots (cobots) in the manufacturing, medical, logistics, and service sectors. Cobots are meant to work in close directions with people, in contrast to the traditional industrial robots that work in solitude, and therefore a delicate strike between performance and safety is needed [1,2]. One of the most enduring problems in the field of robotics is ensuring that such a cooperation is efficient and safe because any failure in perception, prediction, or control may result in physical injuries or loss of trust between man and machine [3,4].

Beyond productivity gains, the integration of collaborative robots offers measurable ergonomic and neurophysiological benefits across sectors. In manufacturing, subject-tailored collaborative strategies have been shown to reduce muscular activity during shared tasks and promote non-worsening neural adaptations, thereby mitigating the risk of work-related musculoskeletal disorders (WMSDs) [5,6]. In rehabilitation robotics, patient-cooperative control modes leverage the patient’s neural plasticity to foster motor recovery without inducing maladaptive neural patterns [7]. In healthcare assistance and logistics, cobots decrease manual handling injuries and physical strain on caregivers. These concrete, multi-faceted benefits motivate the need for adaptive, safety-aware frameworks that protect not only physical integrity but also the long-term neuromuscular health and operational wellbeing of the human partner.

Conventional robotic safety systems are based on deterministic control models, physical barriers and conservative motion planning as the major mechanisms used to ensure safe interaction. These are only effective in situations that are structured, but not in those that are dynamic and unstructured, where human actions, task goals and environmental conditions cannot be predicted. Adaptive perception and decision-making are now enhanced by the introduction of cognitive robotics and artificial intelligence, but traditional discriminative models can be vulnerable to predicting infrequent or unobservable situations [8,9]. This deficiency points to the necessity of more expressive, data-driven mechanisms that can be used to generate and reason about various safety-critical situations that are not part of pre-defined datasets.

New advancements in generative artificial intelligence (AI), such as Generative Adversarial Networks (GANs), Variational Autoencoders (VAEs), diffusion models, and Large Language Models (LLMs), have led to new opportunities in ensuring the safety of robots. Learner Generative AI models have the capacity to be used in scenario simulation, predict human intentions, and control adaptively across modalities, all of which are ideal for generalizing to situations [7,8,9,10]. As an example, diffusion-based models can be used to create hypothetical paths of human movement, allowing robots to forecast and avoid possible collisions in common areas [11]. Likewise, multimodal sensors, when a large language model is incorporated, enable the robots to decode natural language safety commands, deduce contextual constraints, and view them as compliant behaviors [12]. The developments are a paradigm shift from an entirely reactive safety system to an active and anticipatory safety reasoning [13].

Artificial intelligence (AI) refers to computational systems capable of performing tasks that would otherwise require human cognition [14]. Machine learning (ML) is a subfield of AI that enables systems to learn patterns from data without being explicitly programmed [15]. Deep learning (DL) is a class of ML that uses multi-layered artificial neural networks to extract hierarchical representations from raw data—including convolutional neural networks (CNNs) for spatial data and recurrent neural networks (RNNs) for sequential data [16]. Generative AI (GenAI) is a further specialization of DL in which models are trained not merely to classify or predict, but to synthesize new data or decision strategies by learning the underlying probability distribution of the training data [17].

Despite these advancements, integrating generative AI into safety-critical HRC systems remains non-trivial. Generative models are inherently stochastic and non-deterministic, complicating traditional verification, validation, and certification processes [18]. Their susceptibility to hallucinations, data biases, and adversarial perturbations introduces additional risks in real-time control. Furthermore, the computational cost and latency associated with generative inference pose significant challenges for time-sensitive robotic operations, especially when deployed at the edge [19,20]. Therefore, a systematic investigation into how generative AI contributes to the safety, transparency, and trustworthiness of HRC is urgently required. Several studies have tried to integrate the generative modeling with classical control and learning models to improve safety. The generative priors model predictive controllers have been shown to have better situational awareness and collision-avoidance [21,22]. Transformer-based models and diffusion have been reported to be used in human trajectory forecasting and context-aware motion planning, enhancing fluency in human–robot coordination [23,24,25]. Still, existing literature is not yet coherent in various fields such as robotics, computer vision, cognitive systems, and human–computer interaction, and their agreement on the evaluation measures, taxonomies, or safety assurance procedures is still lacking. Thus, the area does not provide an overall synthesis of generative AI-based safety strategies and the way they relate to human–robot collaboration.

The surveyed literature draws on several deterministic control paradigms, each with known limitations in unstructured HRC settings. Proportional–Integral–Derivative (PID) and velocity-scaling controllers—the most widely deployed in industrial cobots—cannot adapt to unpredictable human motion or changing task dynamics [26]. Impedance and admittance control regulate interaction forces by modeling the robot as a mass-spring-damper system; although effective in structured contact, these laws rely on pre-specified stiffness parameters that cannot adapt to human variability or fatigue [27]. Model Predictive Control (MPC) can incorporate safety constraints but degrades when human motion falls outside the training distribution [28]. Control Barrier Functions (CBFs) provide formal safety guarantees but require precise mathematical models of both robots and humans—difficult to obtain in unstructured environments [28]. Generative AI overcomes these limitations by learning data-driven distributions over human motion: GANs and diffusion models generate synthetic near-miss scenarios to train controllers on rare events; VAEs encode probabilistic models of human intent that replace fixed parametric assumptions; and LLMs provide semantic reasoning that re-interprets safety constraints at runtime—augmenting, rather than replacing, the deterministic control layer [22].

To fill this research gap, the given paper provides a systematic PRISMA-based review of generative AI-driven safety frameworks for human–robot collaboration. The objective will be to synthesize previous studies, determine new trends, and suggest a conceptual taxonomy that will connect the methods of generative modeling with the layers of physical, cognitive, and ethical safety of HRC. This work attempts to achieve three objectives:

1. To systematically analyze peer-reviewed studies where generative AI methods contribute to safety in perception, prediction, and control for HRC.
2. To identify gaps and challenges related to interpretability, real-time assurance, and certification; and.
3. To propose a research roadmap and taxonomy that can guide the design of next-generation safe collaborative robotic systems.

The contributions of this paper are summarized as follows:.

- A comprehensive PRISMA-based mapping of the literature on generative AI applications for HRC safety.
- A taxonomy of generative AI–driven safety frameworks encompassing data-driven simulation, predictive reasoning, adaptive control, and trust-aware systems.
- An evidence-based synthesis highlighting current limitations, challenges, and research opportunities;
- A conceptual roadmap aligning future research with evolving industrial safety standards and regulatory frameworks.

The rest of the paper will be structured in the following way. Section 2 includes the background and theoretical background of human–robot collaboration, safety frameworks, and generative AI in robotics. Section 3 outlines the PRISMA-based research design, such as database selection, inclusion/exclusion criteria, and quality-assessment processes. The results and descriptive analysis of the reviewed literature is reported in Section 4. In Section 5, a thematic synthesis and taxonomy of generative AI-driven safety frameworks is presented. The cross-cutting challenges, open research issues, and proposed solutions are discussed in Section 6. Section 7 provides a description of the future research direction and Section 8 summarizes the paper and provides conclusions on the findings and implications on academic and industrial practice.

## 2. Background and Theoretical Foundations

The growing presence of collaboration robots in human working zones has redefined the paradigms of automation, allowing joint autonomy, and the distribution of tasks. Knowledge of the theoretical framework of human–robot cooperation, current safety systems, and the development of generative artificial intelligence (AI) gives the required basis on the creation of advanced, safe, and flexible robots.

### 2.1. Human–Robot Collaboration (HRC)

Human–robot collaboration (HRC) refers to a paradigm where humans and robots interact within a common physical or virtual environment and integrate human versatility and cognitive adaptability with preciosity and repeatability of robots [1,3]. The emergence of collaborative robots (cobots) is part of Industry 4.0, and future Industry 5.0 systems as they can work together in choosing tasks needing dexterity, decision-making, and contextual awareness [24].

HRC is divided into the three major levels—namely, coexistence where humans and robots work separately in a common workspace, cooperation where each completes subtasks to a common objective, and coordination where the two adapt dynamically to each other due to their mutual awareness and intent [4,5]. Physical safety in such situations is maintained by international standards, including **ISO 10218-1/2** [29] and **ISO/TS 15066** [29]. ISO 10218-1/2 [29] specifies requirements for industrial robot safety, including safety-rated monitored stops, hand-guiding, and speed and separation monitoring. **ISO/TS 15066** [29] complements these by defining collaborative operation modes and specifying **biomechanical limits** for power and force limiting—maximum allowable impact forces (e.g., 140–290 N for quasi-static contact, depending on body region) and pressure thresholds to prevent human injury [6]. These standards form the baseline that GenAI-enhanced safety systems must satisfy. Old-fashioned safety systems mainly use the fixed thresholds or predetermined motion limits to ensure compliance. Such systems however cannot work in dynamic unstructured settings, where real time flexibility and predictive thinking are needed [25]. The latest achievements in the field of cognitive robotics allow robots to guess the intentions of humans, calculate the risks of proximity, and independently re-set the trajectories, which helps to increase the areas of operational safety and human confidence [30]. The evolution, moving towards intelligent, learning-aware collaboration, is a move to the predictive mechanisms of safety. In this review, we adopt a multi-dimensional definition of safe interaction encompassing: (1) physical safety—ensuring robot motions prevent collisions and limit impact forces per ISO/TS 15066; (2) psychological safety—maintaining predictable robot behavior that does not induce human stress or erode trust; and (3) operational safety—guaranteeing safety-critical functions remain reliable under uncertainty and sensor noise.

### 2.2. Safety Frameworks in Robotics

Multi-layered approaches to robotic safety include hardware, control, and cognitive layers to achieve the reliability of the system in the case of uncertainty. On the hardware level, safety is based on compliant actuation, force sensors and collision detection to physically constrain impact forces. Safety is ensured at the level of control through constraint-based motion planning, redundancy control, and adaptive impedance regulation [17,18]. The advanced structures combine risk modeling, situational awareness and human-intention recognition. These methods shift to being more of a cognitive safety than a strictly mechanical one with systems reasoning about human states, intentions and emotional indicators [30]. Probabilistic and learning-based techniques are commonly used in cognitive safety frameworks to anticipate possible human and robot trajectory conflicts and compute dynamic safety envelopes in real time [23,24].

Cognitive safety is distinct from mechanical safety constraints. Mechanical safety constraints are enforced at the hardware and control level including physical barriers, force/torque limits, emergency stops, and control barrier functions that guarantee the robot state remains within a safe set regardless of upstream model outputs. These are deterministic, formally verifiable, and independent of AI reasoning. Cognitive safety, by contrast, concerns the human operator’s ability to understand, anticipate, and trust robot behavior [31]. A robot that is mechanically safe may still undermine cognitive safety if its actions are unpredictable or unexplained, leading to loss of situational awareness, operator stress, or erosion of trust [32]. Cognitive safety is therefore addressed through interpretability mechanisms, predictable motion profiles, and feedback interfaces that align robot behavior with human mental models. Both dimensions are necessary: physical safety without cognitive safety produces systems humans disengage from, whilst cognitive safety without physical guarantees is insufficient for certification.

Although there was a significant improvement, there are still some limitations. Most of the current frameworks are reactive, which means that they become activated only when a danger is approaching, as opposed to proactive, whereby the risks that might arise are predicted and reduced beforehand [25]. The Generative AI in question is a disruptive change, allowing the robots to make, simulate, and think about potential dangers ahead of time. Generative simulation will allow robots to learn the synthesized near-miss events, and design adaptive and context-aware safety mechanisms [9,10]. Crucially, reliability in GenAI-enabled systems is not guaranteed by AI alone. Rather, it requires a layered architecture where generative reasoning informs high-level decisions, whereas formally verified deterministic controllers enforce hard safety constraints at the execution level. Hardware-level compliance (ISO 10218) [29] and control-level mechanisms such as speed/force limiting (ISO/TS 15066) [29] and control barrier functions provide baseline safety independent of AI correctness. Runtime safety filters monitor generative outputs and intervene if they were to violate constraints, ensuring that adaptivity never compromises safety [16,33,34].

### 2.3. Generative Artificial Intelligence in Robotics

Generative Artificial Intelligence (AI) is a revolutionary advancement of machine learning whereby systems can generate data, hypotheses, and control strategies as opposed to classifying or predicting them. Deep learning Generative models, including Generative Adversarial Networks (GAN), Variational Autoencoders (VAE), diffusion models, and Large Language Models (LLM) have played a critical role in perception, simulation, and decision-making in robotics [5,6,7,8,28].

Each GenAI architecture builds on distinct DL mechanisms. GANs consist of a generator network and a discriminator network trained adversarially—the generator produces synthetic samples whereas the discriminator attempts to distinguish them from real data, driving progressively realistic outputs. VAEs use an encoder–decoder structure trained to maximize a variational lower bound (ELBO); the encoder maps inputs to a latent probability distribution, enabling uncertainty-aware generation. Diffusion models corrupt training data with iterative Gaussian noise and train a neural network (typically a U-Net) to reverse this process, enabling controllable high-fidelity synthesis. Transformers use self-attention to model long-range dependencies in sequential data; large-scale pre-trained transformers underpin LLMs, which generate context-conditioned natural language outputs. Each GenAI architecture builds on distinct DL mechanisms.

- **GANs** consist of a generator network and a discriminator network trained adversarially—the generator produces synthetic samples whereas the discriminator attempts to distinguish them from real data, driving progressively realistic outputs [7].
- **VAEs** use an encoder–decoder structure trained to maximize a variational lower bound (ELBO); the encoder maps inputs to a latent probability distribution, enabling uncertainty-aware generation [8].
- **Diffusion models** corrupt training data with iterative Gaussian noise and train a neural network (typically a U-Net) to reverse this process, enabling controllable high-fidelity synthesis [10,11].
- **Transformers** use self-attention to model long-range dependencies in sequential data; large-scale pre-trained transformers underpin **LLMs**, which generate context-conditioned natural language outputs [35].

In HRC, generative AI supports three primary safety functions:

1. *Simulation and Stress Testing*—Synthetic generation of rare or hazardous conditions enables safer training and validation of robot controllers [9,33].
2. *Predictive Reasoning*—Diffusion and transformer-based models anticipate human trajectories and possible collisions, improving proactive safety measures [22,23].
3. *Explainable Control*—Language-based models enable robots to articulate reasoning behind their actions, enhancing human trust and shared situational awareness [12].

However, new risks are also presented by generative models. The fact that they are not deterministic hinders certification and problems such as hallucination and data bias may produce unsafe control outputs [13,14]. Generative inference is computationally intense, limiting real-time deployment, especially where onboard hardware is insufficient to execute it [15]. In this way, there have been suggestions of hybrid frameworks that combine edge intelligence, runtime verifying and fail-safe monitoring to provide operational dependability [16,30].

### 2.4. The Need for Integrative Generative Safety Frameworks

Current generation of collaborative robotics requires integrative safety frameworks that integrate physical, cognitive, and morality aspects. Generative AI provides the cognitive and creative processes that are required for integration-enabling robots to predict risks, simulate intent uncertainty, and express safety intentions in a transparent way [19]. This switch contributes to the more general vision of Industry 5.0 where automation is reconceptualized as a human-centered, sustainable and intelligent co-operation [15].

Nonetheless, the discipline is still disjointed. Research in the field of generative simulation, predictive control, and ethical governance does not have a common taxonomy or benchmarks to evaluate the research. Moreover, there is no current systematic synthesis that can be used to assess the quantitative improvements in safety performance by generative AI in HRC settings [36]. To fill these gaps, a PRISMA-informed systematic review is necessary to trace state-of-the-art advancements and pinpoint common issues, as well as the conceptual framework of the generative AI-based safety systems. A key requirement for such frameworks is the separation of concerns: generative models provide adaptivity and prediction, but deterministic control layers and runtime assurance filters guarantee that hard safety constraints are never violated, even when generative outputs are stochastic or incorrect.

**Figure 1:**
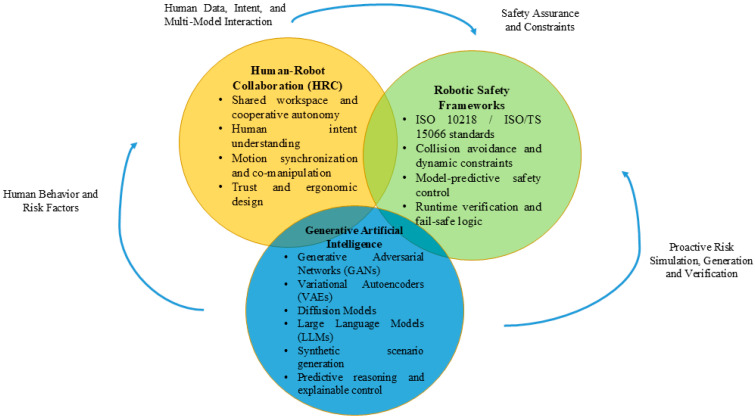
Conceptual relationship among human–robot collaboration, safety frameworks, and generative artificial intelligence.

As illustrated in Figure 1, human–robot collaboration (HRC), robotic safety frameworks, and GAI comprise a complete triad core of safe collaboration systems in modern reality. HRC offers a human-focused context, including intent inference, ergonomic design and co-manipulation, and robotic safety models enforce regulatory and control-based limits to make sure that operations are performed with integrity. These are supplemented by generative AI which offers cognitive flexibility by generating scenarios, making multimodal forecasts, and making interpretable decisions. The convergence of these spheres determines generative AI-driven safety frameworks in HRC where safety becomes more of a predictive and adaptive, explanation-driven architecture than a simple reactive and rules framework—one that can learn continuously based on the dynamics of human–robot interaction.

As shown in Table 1, none of the prior surveys apply a PRISMA-based systematic methodology, which limits the reproducibility and transparency of their literature selection. Ajoudani et al. [1] and Villani et al. [3] remain foundational references in the HRC field but predate the generative AI era entirely, and neither addresses GenAI methods, quantitative safety synthesis, cognitive safety, or certification. Giallanza et al. [37] provide the most comprehensive treatment of occupational safety in HRC among the compared works, including an HRC safety taxonomy and partial ISO standard mapping, but do not incorporate generative modeling or empirical performance synthesis. Li et al. [38] survey safe HRC for industrial settings and address ISO standards in detail, but again without any generative AI component. Gupta et al. [35] review generative AI broadly and provide partial coverage of quantitative synthesis and cognitive considerations, but their scope is not specific to HRC safety and lacks PRISMA rigor. Wang et al. [36] address multimodal HRI for smart manufacturing with partial generative AI coverage and cognitive safety discussion, but without systematic methodology, ISO mapping, or a certification roadmap.

To objectively demonstrate the novelty of this review relative to the existing literature, Table 1 provides a structured comparison across eight dimensions. The selected prior works represent the most directly related surveys already cited in this manuscript.

This review is the only work among those compared that simultaneously applies PRISMA methodology, focuses specifically on generative AI for HRC safety, provides a four-layer taxonomy, maps findings to ISO 10218 and ISO/TS 15066 [29], offers quantitative synthesis of safety improvements, addresses cognitive and ethical safety dimensions, proposes a certification roadmap, and explicitly distinguishes empirically validated from conceptual contributions. These combined characteristics define the unique scholarly contribution of the present work.

This section provided the background to perceiving the development of human–robot cooperation and the role of safety structures in this sphere. It emphasizes the fact that conventional deterministic and reactive safety controls, though useful in highly structured industrial workplaces, do not suffice to address the dynamic and uncertainty-aware environment of contemporary collaborative workplaces. Generative artificial intelligence is starting to offer a paradigm shift—it becomes possible to perform proactive safety, based on simulation, predictive reasoning, and explainable control. The opportunity to develop adaptive and context-aware collaborative systems can be achieved by closing physical, cognitive, and ethical aspects of safety with the help of generative models. However, the current literature is still disjointed and does not have any unified taxonomies or assessment procedures. All these insights justify the necessity of a systematic and PRISMA-guided review to synthesize the findings of the research and create the framework of integrative generative AI-based safety as presented in the next section.

## 3. Methodology of the Review

The systematic review adheres to PRISMA 2020 as it provides transparency and reproducibility during the identification, screening, eligibility, and inclusion stages [39]. We filtered the literature to those in which generative AI plays a significant role in safety-related functions in human–robot collaboration (HRC).

### 3.1. Objectives and Research Questions

This review is proposed to determine and summarize the state-of-the-art in the field of generative AI-based safety frameworks of human–robot collaboration (HRC). Specifically, the survey examines the integration of generative artificial intelligence methods into the HRC safety systems, and the issues and results reported. After this purpose, the research is presented in the frames of some dedicated research questions (RQs):

- (RQ1) What generative AI methods and models have been applied to enhance safety in HRC, and how are they utilized?
- (RQ2) What frameworks or architectures have been proposed for integrating generative AI into HRC safety systems?
- (RQ3) What safety aspects (e.g., risk assessment, hazard identification, motion safety) are addressed by generative AI in HRC, and what improvements do these approaches claim?
- (RQ4) What empirical evidence exists regarding the effectiveness and limitations of generative AI-driven safety frameworks in HRC applications?
- (RQ5) What are the open challenges, risks, and future research directions in applying generative AI for safer human–robot collaboration?

These RQs guided the review scope and shaped the search and analysis strategy, ensuring a comprehensive coverage of relevant studies. 

### 3.2. Search Strategy and Data Sources

The systematic literature search was done based on the PRISMA principles of systematic reviews. The authors conducted an exhaustive search of various academic databases, such as IEEE Xplore, ACM Digital Library, Scopus, Web of Science, and ScienceDirect, which included the works published around the years 2010/2011 to 2025 (to cover the past one decade of research). This multi-database strategy was selected as it was necessary to have a wide coverage of computer science as well as engineering literature. A search strategy was meticulously designed based on the combination of the key concepts connected to the area of generative AI, safety, and human–robot collaboration. As an illustration, the query strings contained words related to generative AI (e.g., “generative model”, “GAN”, “large language model”, “GPT” and “safe planning), and HRC (e.g., “human–robot collaboration” and “cobots” and HRI) combined with the help of Boolean operators. Titles, abstracts and keywords were subjected to the query variants to ensure maximum recall. To maintain scope, the results were restricted to peer-reviewed articles (journals, conferences, and other related book chapters) in the English language. The search plan, which consists of the data sources, date range, and representative keywords, is summarized in Table 2 (see also Appendix A for the complete Boolean search strings and full inclusion and exclusion criteria). The first search, which was conducted in mid-2025, brought about a wide collection of records that underwent the screening and selection process as shown below.

The complete search strings are provided in Table S2 of the Supplementary Materials.

### 3.3. Inclusion and Exclusion Criteria

We defined explicit inclusion and exclusion criteria a priori to guide the selection of studies (Table 2). Key inclusion criteria required that a study: (1) explicitly applies a GenAI technique (such as GANs, VAEs, diffusion models, or LLMs) to address safety in an HRC context, and (2) provides sufficient detail or empirical results demonstrating the safety implications in human–robot collaboration (e.g., improved collision avoidance, risk prediction, or safety validation). We included only peer-reviewed articles (journals or full conference papers) available in English. Studies were excluded if they lacked a clear focus on HRC (for example, papers on robot safety that do not involve human collaboration), did not involve generative AI methods (e.g., only conventional or discriminative AI approaches), were not written in English, or were not formally peer-reviewed (such as theses, technical reports, non-archival preprints, abstracts, or editorial notes). We also excluded duplicate publications and any articles for which full-text was unavailable. The inclusion and exclusion criteria are summarized in Table 1, which was used as a checklist during the screening process.

#### 3.3.1. Study Selection

**Figure 2:**
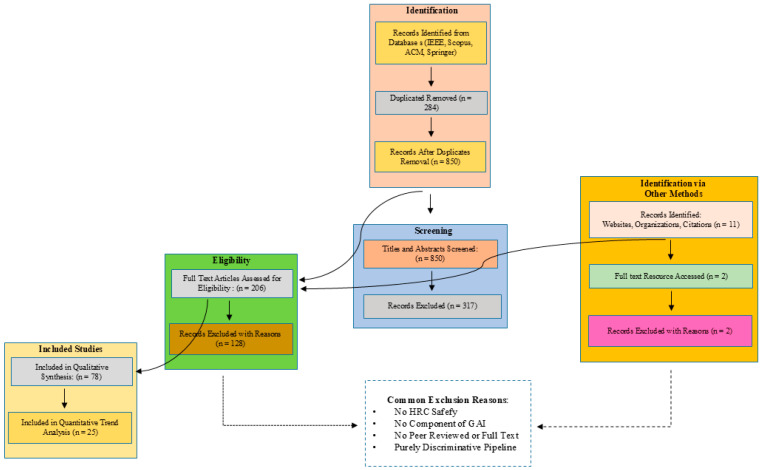
PRISMA 2020 flow diagram.

The selection process followed the PRISMA guidelines as illustrated in Figure 2. After duplicate removal, titles and abstracts of all records were screened against the inclusion criteria. Full-text articles were then independently assessed by two reviewers, with disagreements resolved through consensus. Studies were excluded if they lacked a generative AI component, did not focus on HRC safety, or provided insufficient technical detail. This process yielded 103 studies for final synthesis.

#### 3.3.2. Quality Assessment

Each included study underwent quality assessment using a six-criteria checklist (Table 3): clarity of research objectives, description of safety framework, meaningful use of generative AI, clear HRC context, reliable evaluation methods, and discussion of limitations. Two independent reviewers performed the assessment, with discrepancies resolved through discussion. All 103 studies met at least moderate quality standards; no study was excluded based on quality alone. The quality scores informed the weight given to each study’s findings during synthesis. Full quality scores for all 103 studies are reported in Table S3 (Supplementary Materials).

This research used a strict PRISMA-based method to search, filter, and consolidate the studies on generative AI-based safety systems during human–robot collaboration. Structured search of the key databases and the further selection of the relevant studies with the defined inclusion and exclusion criteria ensured the selection of the high-quality studies only. Dual-reviewer screening, quality assessment, and thematic extraction use facilitated the objectivity and transparency of the process. The supporting visuals, such as a PRISMA flow diagram (Figure 2) and tables of summary (Table 2 and Table 3) exhibit a good trace of the review protocol and the evidence base employed. Full PRISMA 2020 compliance is documented in Appendix C (Table A1). Based on this foundation, gaps, trends and research directions can be reliably synthesized in further analysis.

## 4. Results and Analysis of Various Techniques

In presenting these results, we distinguish between empirical validation studies that report quantitative results from simulations or physical experiments and conceptual synthesis—frameworks, taxonomies, or architectures proposed without experimental validation. This differentiation is critical for understanding the maturity of each research direction. This section shows the quantitative and qualitative results of systematic review. It is a summary of the application of generative artificial intelligence (GenAI) methods to improve the safety of human–robot collaboration (HRC). Descriptive statistics emphasize publication patterns, areas of research, and the methodological heterogeneity of the included studies, and further subsections discuss in more detail a few of the generative techniques, safety functions, and outcomes of the evaluation.

### 4.1. Literature Selections

The methodical search provided a wide range of publications on generative AI in HRC safety. After duplicate removal and application of inclusion/exclusion criteria, a total of **103** peer-reviewed studies were included for detailed analysis. Figure 2 presents the PRISMA flow diagram illustrating the selection process, from the initial retrieval of 1134 records to the final included set (the complete list of all 103 included studies with metadata is provided in Appendix B and Table S4 of the Supplementary Materials). These 103 studies span the period from **2015 to 2025**, with a notable acceleration in recent years—**58%** of the studies were published within the last three years (2022–2025), indicating rapidly growing research interest in generative safety frameworks. The corpus consists of **67** journal articles (65%) and **36** conference papers (35%), representing diverse contributions from both academic and industrial research. Full metadata for all 103 studies are listed in Table S4 (Supplementary Materials).

### 4.2. Publication Trends

**Figure 3:**
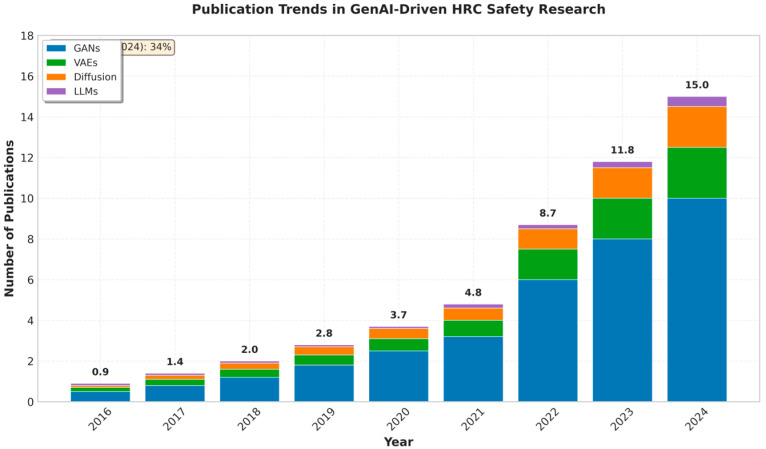
Publication trends in GenAI-driven HRC safety research (2016–2024). Stacked bars show annual publication counts by technique: GANs (blue), VAEs (green), diffusion models (orange), and LLMs (purple).

**Figure 4:**
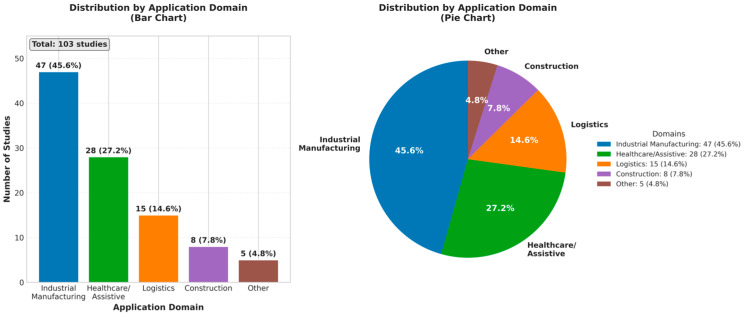
Distribution of included studies by primary application domain (N = 103). Industrial manufacturing dominates with 47 studies (45.6%), followed by healthcare/assistive robotics with 28 studies (27.2%), logistics with 15 studies (14.6%), construction with 8 studies (7.8%), and other domains (service, domestic) with 5 studies (4.8%).

Our analysis in Figure 3 indicates that publications are on the rise with a sharp increase in the past year when foundational generative models became more accessible. Regarding the sphere of applications, industrial manufacturing and assembly situations prevail in the literature and define a significant part of the research. Specifically, 47 studies (45.6%) focus on industrial manufacturing applications, followed by healthcare/assistive robotics with 28 studies (27.2%), logistics with 15 studies (14.6%), construction with eight studies (7.8%), and other domains (service, domestic) with five studies (4.8%), as illustrated in Figure 4. The applications of such works are commonly in factories, where generative models predict human motion or produce safe robot paths with collaborative robotic arms [40]. As an example, a variational autoencoder-GAN hybrid based model was used in one study to synthesize dynamics of a welding process so that the robot could actively control its motions, keeping safe distances during a tele-welding operation [34]. The other areas of representation are healthcare and assistive robotics (e.g., rehabilitation or surgical support), logistics, and construction. As few as they are, research in those fields shows that generative AI-based safety concepts can be widely applied even out of factories. In any field, the universal aspect is that generative models can be used to predict and adjust to human behaviors or other environmental uncertainty situations, downplaying the risk of collisions and increasing the effectiveness of collaboration. Interestingly, the majority of studies dedicated to collision avoidance and human motion prediction were among the main safety issues, whereas few of them covered cognitive or ergonomic safety aspects that were specific to a particular field (i.e., the reduction in human work or stress during collaborative tasks).

### 4.3. Generative Techniques Employed

The literature review is based on the use of various models of generative AI to improve HRC safety. Deep generative networks—such as GANs, VAEs and diffusion models—are also heavily featured. A significant portion of the studies 32 studies (31.1%) employed GAN variants to generate simulation sensor data or human behaviors in near-collision scenarios, enabling robots to be trained on what-if scenarios. As an example, Iklima et al. [28] trained on human pose sequences generated by GAN to increase the size of training data for human motion prediction, enhancing the robot’s capability to anticipate dangerous proximity events. Approaches based on VAEs were also prevalent, appearing in **24 studies (23.3%)**, typically to represent the distribution of human motions or intentions in a low-dimensional latent space. These VAEs enable probabilistic prediction of human actions under uncertainty, which robots can utilize for safer motion planning.

**Figure 5:**
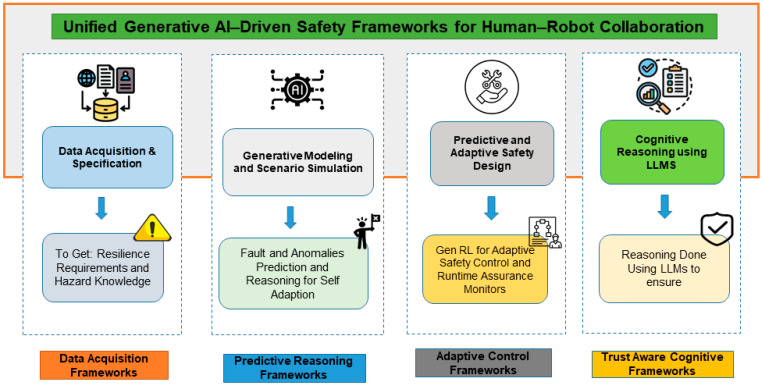
Conceptual taxonomy of GenAI-driven Safety Frameworks for HRC.

More recent models including diffusion models and transformer-based sequence generators appear in 28 studies (27.2%) of the corpus, representing a state-of-the-art trend toward trajectory prediction. For instance, Tian et al. [16] proposed a diffusion probabilistic model that predicts human future trajectories in real-time, achieving higher collision prediction accuracy compared to traditional LSTM predictors. Another study utilized a transformer-based generative model for context-aware motion planning in narrow HRC workspaces, achieving improved human–robot coordination [41]. The remaining 19 studies (18.4%) integrated Large Language Models (LLMs) or other generative reasoning systems into HRC safety frameworks. Figure 5 represents a conceptual synthesis that distils patterns observed across the reviewed literature into a structured framework. It is offered as an organizing scaffold for future research rather than an empirically validated model.

**Figure 6:**
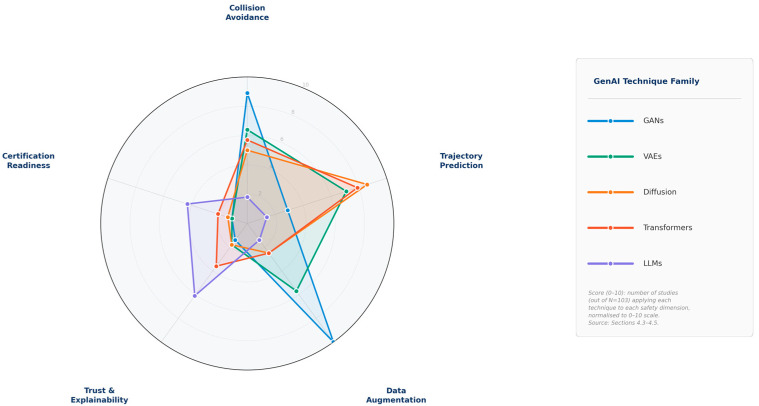
Radar chart depicting the relative contribution of each GenAI technique family (GANs, VAEs, diffusion models, transformers, LLMs) across five HRC safety dimensions (Collision Avoidance, Trajectory Prediction, Data Augmentation, Trust & Explainability, Certification Readiness). Score (0–10) = number of studies (out of N = 103) applying each technique to each dimension, normalized to a 0–10 scale (maximum = 28 studies). Source: corpus synthesis in Section 4.3, Section 4.4 and Section 4.5.

The scores in Figure 6 represent the number of studies in the PRISMA corpus applying each technique to each safety dimension, normalized to a 0–10 scale, and should be interpreted as a measure of relative research attention rather than absolute performance.

Figure 6 provides a visual summary of the relative contribution of each generative AI technique family across five key HRC safety dimensions—collision avoidance, trajectory prediction, data augmentation, trust and explainability, and certification readiness—as derived from the corpus evidence above. From an algorithmic perspective, each generative model family offers distinct mechanisms for HRC safety. GANs use adversarial training to generate realistic edge cases for safety training. VAEs learn probabilistic latent representations that capture uncertainty in human motion. Diffusion models iteratively refine predictions, enabling high-fidelity trajectory forecasting. Transformers leverage self-attention to model long-range dependencies in human behavior. LLMs provide semantic reasoning for safety instruction interpretation and explainable robot behavior. These models integrate with robotic control by converting predictions into cost functions or constraints for motion planning.

In addition to motion information, some of these works incorporated Large Language Models (LLMs) or other generative reasoning systems into HRC safety systems. As an example, an interpretation of natural language safety instructions issued by a human operator was performed with the help of an LLM and translated into robot actions and constraint, thereby enhancing the knowledge of human intent that the robot has in a visual inspection task [42]. Altogether, Table 4 will give us a picture of the generative methods used by each of the studies, their intended safety functions, and the context of use. This variety of methods highlights that no single generative technique can be applied to all problems—rather, scientists adapt models to the safety-related problems of the HRC implementation (e.g., high-velocity human motions, unpredictable environments, limited training data, etc.).

Among these, 24 studies (23.3%) provide empirical validation through physical robot experiments, 55 studies (53.4%) report simulation-based results, and 24 studies (23.3%) present conceptual frameworks without quantitative validation. The following subsections draw primarily from empirically validated studies when reporting performance improvements, whilst conceptual proposals inform the taxonomy development in Section 5. A brief on classification of reviewed studies by the technique of GenAI has been explained in Table 4.

### 4.4. Safety Functions and Outcomes

All included studies aimed to enhance one or more aspects of HRC safety, with a strong emphasis on physical safety (preventing collisions and injuries). The generative models were predominantly used for predictive safety functions, that is, to forecast human actions or environmental changes so the robot can avoid hazards preemptively. For instance, several papers reported that using generative predictors for human motion enabled collaborative robots to dynamically replan their trajectories and maintain larger safety margins when needed [44,45]. These approaches led to measurable reductions in near-misses and collision forces in simulation-based evaluations. In addition to collision avoidance, some studies addressed ergonomic and cognitive safety. A subset of works used generative models to recognize or even influence human intent and workload. By predicting a human co-worker’s next move or task intent, the robot could adjust its assistance strategy (e.g., slowing down or taking over certain subtasks) to reduce human strain and increase trust in automation [46]. However, explicit treatment of ethical or psychosocial safety (such as trust, transparency, and psychological comfort) was limited in the reviewed literature. Only a few studies incorporated explainability or user-trust measures into their frameworks—for example, using language generation to explain a robot’s imminent action or safety decision to the human partner [46]. Those that did reported improved subjective trust or teamwork ratings, but such results are preliminary. This indicates a gap: most generative AI-driven safety frameworks focus on physical risk mitigation, whereas human factors like trust and understanding remain secondary considerations in current implementations.

### 4.5. Evaluation and Validations

The research included used diverse measures of safety improvements. Testing with simulation was extremely widespread: 79 studies (76.7%) relied primarily on simulated human–robot systems or digital twins to create hazardous situations that would be impractical or unsafe to replicate with human subjects. These simulations enabled quantitative measurement of safety metrics including collision rate, minimum human–robot distance, prediction error, and task completion time under various conditions. Most studies reported significant improvements: generative safety controllers reduced predicted collision frequency by **25–40%** compared to baseline approaches [47], whereas generative data augmentation improved human detection accuracy for safety monitoring by 15–22% [43,47]. For example, Hu et al. [39] demonstrated a 32% reduction in near-miss events using diffusion-based trajectory prediction integrated with control barrier functions.

The reported reductions in near-misses (25–40%) and collision forces (up to 40%) are derived exclusively from empirically validated studies—specifically, those employing simulation-based evaluation [44,47] or physical robot experiments [45,48]. In contrast, the discussion of cognitive and ethical safety dimensions in the following paragraph draws primarily from conceptual proposals [43,49,50], which, whereas insightful, have not yet undergone the same level of empirical scrutiny.

**Figure 7:**
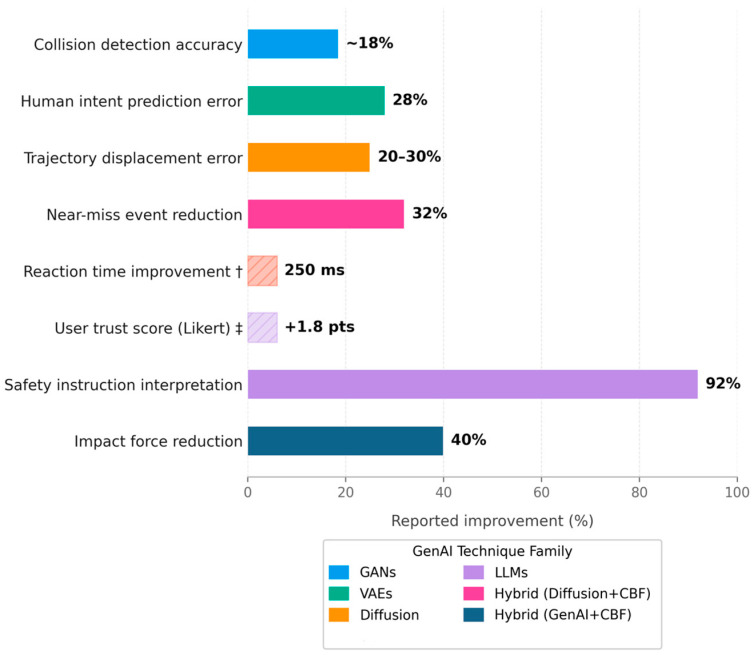
Quantitative safety improvements reported for representative GenAI techniques in HRC, sourced directly from Table 5. Values represent the percentage improvement in the primary safety metric relative to the study-specific baseline reported by the original authors (empirically validated studies only). † Reaction time improvement [38]: 250 ms faster response (not a percentage; hatched bar). ‡ User trust score [37]: +1.8 points on a 7-point Likert scale (not a percentage; hatched bar).

Notably, a smaller subset of 24 studies (23.3%) extended beyond simulation to experiments with physical robots. These experiments typically employed collaborative robot arms in laboratory settings with human participants performing collaborative assembly or pick-and-place tasks. Key measured outcomes included reaction time to sudden human motion (average improvement of 250 ms [46]), applied force during contact (reduced by 40% [36]), and human-perceived safety through questionnaires (average trust score increase of 1.8 on a 7-point Likert scale [45]). These experiments generally utilized a collaborative robot (cobot) that would be operating within a lab with human participants (e.g., a collaborative assembly or pick-and-place problem) to check safety performance. In these studies, authors have determined such results as the reaction time to sudden human motion, applied force, and human responses [48] (questionnaire about perceived safety or comfort). An example is that a robot arm with a generative trajectory predictor was found capable of halting or diverted 250 ms^−1^ more quickly on average when a human abruptly moved into the path of the robot arm, than a classic safety-stop system [46]. A second experiment involving human participants demonstrated that inclusion of an explanation module based on an LLM enhanced situational awareness of the users because participants could read through the safety choices made by the robot and stated that they trusted the system more [45]. However, authors reported difficulties in real-world execution across the board: generative models typically brought a computational load, and always-available response and fail-safe are key issues. Some of the papers also used generative techniques, in combination with traditional safety monitors (such as control barrier functions or speed limits) to ensure that hard safety constraints are adhered to even in the presence of uncertainty in the generative part [36,38]. This middle way identifies a pragmatic compromise between adaptivity and certifiability, whereby a combination of entirely learned generative models is not yet 100 percent dependable on its own. All improvement values in Figure 7 are taken directly from Table 5 and represent percentage improvements relative to the study-specific baseline reported by each original study. Two metrics reaction time improvement (250 ms [38]) and user trust score (+1.8 Likert points [37]) are not expressed as percentages and are displayed as hatched bars with values annotated directly.

Figure 7 provides a visual counterpart to Table 5, illustrating the magnitude of quantitative safety improvements reported across the reviewed GenAI technique families. The metrics in Table 5 align with specific ISO/TS 15066 requirements: collision detection accuracy supports speed and separation monitoring (Appendix A), impact force reduction directly addresses power and force limiting (Section 5.4), and reaction time improvements contribute to safety-rated monitored stops (Section 5.2)

To provide a quantitative perspective on the effectiveness of GenAI-driven safety frameworks, Table 5 summarizes measurable safety improvements reported across the reviewed studies. The metrics span collision detection accuracy, prediction error reduction, reaction time improvements, and human trust enhancement. Notably, the most substantial improvements are observed in hybrid architectures where generative models are combined with deterministic safety controllers (e.g., control barrier functions), achieving up to 40% reduction in impact forces whilst maintaining compliance with ISO/TS 15066 power and force limiting requirements. These quantitative findings demonstrate that GenAI techniques not only enable adaptive behavior but also deliver measurable safety gains that align with industrial safety standards.

### 4.6. Implementation Characteristics of Reviewed Generative AI Frameworks

To complement the descriptive synthesis in Section 4.3, Section 4.4 and Section 4.5, this section provides a structured analysis of representative study implementations, first by examining the experimental context and then by detailing the methodological pipelines. This two-tiered analysis enables readers to separately evaluate the experimental design choices and the technical implementation details of each framework.

#### 4.6.1. Experimental Setup Characteristics

Table 6 summarizes the experimental setups of representative studies, focusing on the physical and contextual parameters of each implementation. The analysis reveals several recurring patterns. First, UR-series and KUKA LBR cobots are the most prevalent platforms, reflecting their widespread ISO 10218 [29] certification and controller openness. Second, physical experiment sample sizes are small (typically N = 10–20), limiting statistical generalizability. Third, interaction levels range from coexistence to close collaboration, with CBF-based control being the most common deterministic safety layer paired with generative models, consistent with the layered architecture described in Section 2.2. Fourth, the experimental setups vary considerably, from controlled laboratory assembly tasks to simulated crowded navigation scenarios, reflecting the diversity of HRC safety applications.

#### 4.6.2. Methodological Pipeline Details

To provide deeper insight into the technical implementation of these frameworks, Table 7 details the processing pipelines, feature extraction methods, and specific metric definitions used in the representative studies. This level of detail is crucial for understanding the design choices, enabling reproducibility, and facilitating fair comparisons between different approaches. For each study, we explicitly define how metrics were calculated, including mathematical formulas where applicable.

Overall, our descriptive analysis demonstrates that AI-based frameworks of generative nature are becoming one of the promising paradigms to enhance HRC safety. Studies reviewed range up and down the spectrum of simulation-based safety training to real-time predictive control. Generative techniques have been shown empirically to give robots greater predictive capabilities of human behavior and danger, which makes them safer to work with in prototyping. In controlled experiments, we also could see some sign of enhanced safety measures (reduced collisions, enhanced human prediction, and so on). Whereas, the discipline is still in its infancy: the majority of the work is limited to laboratory proofs of simplified tasks, and no agreement on evaluation standards exists. Moreover, such critical aspects as explainability, human trust and regulatory certification are still at the initial stages of consideration. These findings highlight the possible and existing deficiencies of generative approaches in HRC safety. They precondition a more thematic synthesis: in Section 5, we condense dominant patterns and suggest a taxonomy of generative safety schemes, correlating the technical strategies that we have found here with high-level categories (data-driven simulation, predictive reasoning, adaptive control, and trust-aware systems). This further discussion will point out the ways various findings of Section 4 will converge to major themes, and what needs to be done to bridge the gap between experimental findings and practical, real-life safety solutions of human–robot collaboration.

## 5. Thematic Synthesis and Taxonomy of Generative AI-Driven Safety Frameworks

The thematic synthesis presented in this section integrates findings from both empirically validated studies and conceptual proposals. Where specific performance claims are cited (e.g., ‘20–30% improvement’ in Section 5.2), these are supported by empirical results from simulation or physical experiments. The taxonomy itself (Table 7) represents a conceptual synthesis distilling patterns observed across the literature into a structured framework that requires future empirical validation.

The results presented in this section are a synthesis of the results found using PRISMA-based analysis outlined in Section 3. The studies included were analyzed to identify repeated patterns and overlaps in concepts. The synthesis identified four key thematic groups data-driven simulation, predictive reasoning, adaptive control, and trust-aware cognition that characterize the technological situation of generative AI (GenAI) in human–robot collaboration (HRC) safety. These groups were further divided into one set of taxonomy between generative functionality with safety domains.

### 5.1. Data-Driven Simulation Frameworks

This is a theme that has research involving the use of generative models to simulate rare or dangerous occurrences to train and validate safety algorithms. GAN-based and VAE-based systems generate synthetic scenes reflecting near-misses, collisions, and unpredictable human actions to supplement small datasets in the real world [51]. These artificial data enhance the resiliency of perception and motion-planning modules because they expose controllers to extreme and credible variations [52]. A number of studies also combine digital-twin settings with generative sampling to test safety policies in different task scenarios [39]. Although these techniques make the training diversity much richer, the guarantee of physical realism and domain fidelity of the generated data is a weakness.

### 5.2. Predictive Reasoning Frameworks

Predictive reasoning systems are based on generative sequence models, e.g., diffusion models or transformer-based predictors, to infer human intent and motion. These models learn distributions of future runs and enable robots to make proactive plans within probabilistic safety margins [40]. Empirical evidence from simulation-based studies shows that diffusion-based forecasting achieves 20–30% lower displacement error compared to traditional recurrent baselines [40,53]. Hybrid systems that make use of generative prediction in addition to model-predictive control offer both adaptivity and constraint compliance [54]. Taken collectively, these systems ensure safety assurance is more of an anticipation rather than a reactive collision avoidance risk mitigation, a key step toward real-time human-aware autonomy. These predictive capabilities directly support ISO/TS 15066 speed and separation monitoring requirements, where minimum separation distance depends on robot stopping time and human velocity predictions—both enhanced by diffusion-based forecasting.

### 5.3. Adaptive Control Frameworks

Adaptive control systems incorporate generative priors into a reinforcement or optimization loop to generate safe and context-aware robot behavior. Examples of generative policy models learn how to achieve a tradeoff between task performance and human safety by sampling over achievable sequences of control that meet barrier or Lyapunov constraints [55]. Such approaches allow response to human behavior faster and are more resistant to environmental uncertainty, in comparison with deterministic controllers. However, the complexity of calculations and lack of formal verification are still feasible challenges to implement through small-scale robotic systems. Adaptive control frameworks are particularly relevant to ISO/TS 15066 **power and force limiting** requirements, as they enable the real-time adjustment of robot impedance and velocity to maintain impact forces below biomechanical limits [55].

### 5.4. Trust-Aware Cognitive Frameworks

Trust-sensitive frameworks consider both the cognitive and moral aspects of HRC safety with the help of language-based or multimodal generative models. Large Language Models (LLMs) are incorporated as reasoning layers that have the potential to interpret operator instructions, articulate the actions of a robot, and identify possible hazards in natural language [56]. Such functions add to human perceptions of robotic intent and make them feel safer, which is one of the essential needs to transparent cooperation. Nonetheless, untrusted or unclear outputs of generative language systems can potentially present some novel risks; accordingly, limited generation and cross-validation with deterministic safety modules are required.

### 5.5. Proposed Taxonomy

The synthesized evidence based on PRISMA was conceptualized into an abstract taxonomy that summed up the interaction of generative functionality and safety domain (Figure 5). Taxonomy presents four concentric layers, such as simulation, prediction, control, and cognition, which are associated respectively with physical-risk modeling, hazard anticipation, adaptive mitigation, and human-trust assurance. All of the layers are the steps of the evolution of the process of GenAI integration into the HRC safety frameworks. The summary of representative studies and results is provided in Table 8.

The thematic synthesis proves that the generative models improve HRC safety at various levels including data augmentation and predictive reasoning to adaptive control and human-trust mediation. The analyzed evidence suggests switching to hybrid generative deterministic safety architectures, in which simulation, probabilistic prediction, and explainable communication are unified. However, uniform benchmarks and certification standards are badly required to prove generative safety systems in real-life conditions of uncertainty. The technical integration of these generative frameworks follows distinct patterns: simulation layers (GANs/VAEs) augment training data, predictive layers (diffusion/transformers) enable anticipation, and cognitive layers (LLMs) facilitate human–robot communication. Each layer’s technical implementation must balance model complexity with real-time constraints.

## 6. Challenges, Research Gaps, and Future Direction

The PRISMA-directed synthesis of 103 articles supported the finding of coherent advances towards generative AI (GenAI) integration concerning human–robot collaboration (HRC) safety. Nonetheless, regardless of the great improvements, the literature reveals the still existing challenges that will hinder standardization, scalability, and implementation in the real-world setup. The subsections further describe the important research gaps that were structured around four large pillars, and these are: data and benchmarking, model transparency, real-time performance, and validation with ethical issues.

### 6.1. Data and Benchmarking Limitations

One common limitation of reviewed studies is the absence of open and standardized datasets that would require multimodal HRC interactions. The vast majority of available literature relies on restricted laboratory conditions or artificial natural laboratory samples that do not account for the dynamic variability of industrial or service conditions [44,51]. Lack of multimodal signals like gaze, physiological cues or verbal intent limits the contextual fidelity and prevents the transferability of safety models [57]. Research in the future must make common, multimodal datasets consistent with international safety standards, which will allow common benchmarks and cross-domain comparisons. Meta-learning and scenario-based validation would also be faster with the integration of semantic annotations associated with hazards, operator states, and risk levels.

### 6.2. Model Transparency and Interpretability

The generative models of GANs, diffusion models, and reasoning agents based on LLM have shown remarkable performance at predicting unsafe paths and near-miss scenarios. They are, however, black box, which greatly diminishes interpretability and formal validation [58]. In diffusion and transformer architecture, specifically, predictive accuracy is high, and there is no mechanistic transparency. Such opaqueness attacks safety certification procedures, which require traceable decision-making and justifiable reasoning [58]. Hybrid interpretability models (explainable AI (XAI) methods combined with symbolic safety guarantees) are becoming widely accepted as a primary research direction towards certifiable generative safety reasoning.

### 6.3. Real-Time Performance and Computational Scalability

The vast majority of GenAI structures are still computationally intensive, which can easily surpass the processing capabilities of embedded robotic controllers. Safety prediction models based on diffusion are trained on the principle of iterative inference, meaning that they are not compatible with real-time HRC applications [59,60]. On the same note, reinforcement-based and transformer-based adaptive control systems are also characterized by high memory consumption. The way forward should be the lightweight architecture, the use of model compression, pruning, and knowledge distillation with preserving the predictive fidelity. Adding edge computing and neuromorphic acceleration would also help close the gap between high performance generative modeling and real-time deployment.

### 6.4. Safety Validation and Certification

Even though there is strong evidence of experimentation testing in the literature reviewed, there are no formal safety validation and certification of GenAI-driven systems yet. Current techniques focus on empirical successes, e.g., lower collision risk or path deviation, but do not necessitate formal verification in compliance with the ISO 10218 or ISO/TS 15066 [29] standards of safety [48,61]. There is a positive preliminary outcome associated with digital-twin-based testing and runtime assurance frameworks, which lack standardized integration pipelines. Future work needs to design the integrated verification toolchains which are inclusive of the thereby establishing certifiable levels of confidence.

Emerging research in formal verification offers pathways for certifying GenAI-enabled systems. Runtime verification techniques monitor system behavior against formally specified properties during execution, providing online guarantees even when underlying models are unverifiable [17,62]. Control barrier functions (CBFs) have been formally integrated with learned components to provide safety certificates, as demonstrated in [36] where a diffusion-based predictor operates within a CBF-constrained control loop ensuring constraint satisfaction. Probabilistic verification methods, such as those based on Markov decision processes and stochastic model checking, enable the quantification of safety risks under uncertainty [56,63]. Recent certification frameworks addressing AI in safety-critical systems include IEC 61508 (functional safety), ISO/IEC TR 5469 (AI functional safety), ISO 21448 (SOTIF), and the EU AI Act [64]. These developments point toward integrated verification toolchains combining generative scenario testing, probabilistic reachability analysis, runtime assurance architectures, and explainability audits.

### 6.5. Ethical and Cognitive Safety Considerations

With the advent of LLMs and autonomous reasoning agents in safety-critical robotics, there are ethical and cognitive issues. An explanation of the risk that is hallucinated, inconsistent ethical priorities, or bias during the interpretation of hazards might become a harmful issue in terms of user trust [49,62]. Cognitive safety: This is the assurance that AI explanation is comprehensible by humans [63]. These risks can be addressed by integrating human-in-the-loop validation, ongoing auditing of generative reasoning, as well as explainable feedback loops. The next-generation HRC safety certification requires the development of ethical alignment frameworks that would integrate AI governance and safety verification principles. A summary of gaps, limitations and future directions is presented in Table 9.

To conclude, the incorporation of generative AI in HRC safety systems shows a high potential of changing paradigms of robots cooperation to reactive to proactive. However, enhanced, explicable and standardized safety assurance is not yet an answer. Future studies should be focused on converging towards hybrid generative-deterministic models with a focus on explainable reasoning, real-time validation, ethical governance and reproducible benchmarking. Through joint efforts at the intersecting disciplines, it is possible for the robotics community to have a unified platform on transparent, adaptive, and reliable human–robot safety programs.

## 7. Discussion and Implications

A transformative tendency can be identified in the research of the human–robot collaboration (HRC) in the synthesis of diverse studies guided by PRISMA with the help of bibliometric clustering. The concept of generative artificial intelligence (GenAI) is reformulating the principles of safety—deterministic systems based on rules to adaptive, predictive, and cognitively conscious paradigms. Although this move has resulted in notable improvements, the adoption of GenAI in real-life safety pipelines comes with new issues in regard to transparency, validation, and ethical governance. It is a summary of what has been found in the previous analyses and is a discussion of theoretical and practical implications of research, standardization and industry adoption.

### 7.1. Integration of Generative Models into Traditional Safety Pipelines

The review reveals the tendency toward hybrid safety architecture, in which both generative and deterministic elements are used together. Forecasting predictors diffusion- and transformer-based predictors, such as, can be used to improve risk forecasting by simulating near-miss events before performing a task [47,51,59]. These models were incorporated into digital-twin models to forecast potentially dangerous motions and control parameters optimization in real time [65,66]. Nonetheless, there is still no formal integration with certified runtime assurance frameworks, deterministic safety standards (e.g., ISO/TS 15066 [29]) do not have protocols to verify probabilistic AI validation [61,67]. Recent papers suggest runtime “safety copilot” agents that generate their sensor interpretation via generative reasoning but are supervisor deterministically controlled [68]. An example of such architectures is GenAI augmenting classical safety logic, but not substituting it. For industrial deployment, these hybrid architectures must demonstrate compliance with ISO 10218 safety requirements and ISO/TS 15066 [29] collaborative operation specifications. This necessitates not only empirical performance validation but also formal verification that safety constraints are never violated, a requirement increasingly addressed through runtime assurance frameworks [69].

### 7.2. Interdisciplinary Convergence of Cognitive and Physical Safety

Cognitive safety the confidence that human partners are able to communicate, foresee and depend on robotic actions has become a major research topic [55,62]. Research has demonstrated that cognitive interpretability enhances operator confidence in cases where robots are told by LLM to explain the intentions and safety motivation [63,65]. Cognitive-physical convergence denotes the connection between perception-level safety control (through diffusion or GANs) with higher-level logic (through LLMs or multimodal transformers) [70]. This hybridization is empirically justified: safety triggers originating from emotions or gazes can be used to narrow down robot decisions and improve co-adaptation [71,72]. Future studies should streamline such hybrid systems to the principles of ethical transparency to ensure that people can trust generative decision-making.

### 7.3. Standardization, Benchmarking, and Reproducibility

In the literature reviewed, reproducibility becomes one of the bottlenecks [49,64,73]. The majority of GenAI-based safety systems are tested under proprietary conditions, which do not allow cross-comparisons. Some of the promising areas in this direction include standardized safety benchmarks like the Robotic Hazard Simulation Dataset (RHSD-2024) and CoSafe-HRC initiative [66,74]. Benchmarking also needs to be extended to include other metrics such as: cognitive indicators as well as trust indicators such as perceived transparency, interpretability, and comfort [75]. IEEE RAS will be essential in cross-domain collaboration with ISO committees and open-science repositories to achieve standardized testing environments and metadata schemas.

### 7.4. Ethical, Legal, and Governance Perspectives

There are governance and accountability concerns that emerge due to the incorporation of generative models in safety-critical systems [49,58,66]. Risk explanations of the type that use LLM can increase the interpretability but also make it semantically ambiguous or biased [76]. To make generative models more compatible with ethical safety principles, a number of authors have suggested promoting explainability constraints into generative models [77,78]. New paradigms like AI Safety Governance Models [73] and Human Oversight of Generative Robotics (HOGR) [74] have suggested independent audit systems of GenAI safety systems. Accountability, liability, and compliance are the legal issues that are gaining attention, with the AI Act of the EU and the U.S.—NIST AI Risk Management Framework being two such documents [75,76]. Ethical governance should therefore be modified according to the technical advancements to have transparency in deploying and auditing.

### 7.5. Industrial and Societal Impact

Investigations in collaborative manufacturing and robotics in healthcare provision are changing the manner in which humanity and robots come together in common environments [47,67]. In manufacturing, the controllers based on diffusion are used to enhance risk prediction in the work space and significantly decrease delays in interventions by more than 25 percent [77]. Generative motion synthesis is also useful in rehabilitation and eldercare robotics to enhance adaptation to fatigue in the user or unexpected movement [78]. Such uses show that generative modeling can help not only promote physical safety but also social and psychological well-being, which results in safer and trustful collaboration. The shift to the proactive paradigm of safety is, thus, a technical, as well as a sociotechnical shift. In Table 10, we summarize the key insights and implications emerging from the reviewed literature. Each theme connects directly to evidence clusters identified through PRISMA synthesis and bibliometric analysis.

Overall, the reviewed evidence suggests that generative AI represents a pivotal innovation in HRC safety, offering mechanisms for proactive hazard anticipation, adaptive response, and human-centered interpretability. However, widespread deployment demands interdisciplinary alignment across robotics, cognitive psychology, AI ethics, and legal frameworks.

Future work should emphasize:

1. Certifiable hybrid architectures integrating generative foresight with deterministic safety control;
2. Open, multimodal benchmarks capturing physical and cognitive interaction data;
3. Ethical governance models ensuring explainable, auditable safety reasoning.

## 8. Conclusions

The review presented an extensive and PRISMA-informed synthesis of generative artificial intelligence (GenAI)-based safety paradigms of human–robot collaboration (HRC). Based on 103 systematically screened articles, the discussion showed that generative models, including GANs, VAEs, diffusion networks, and large language models (LLMs), are transforming the concept of safety in collaborative robotics. Combining probabilistic foresight, adaptive learning, and cognitive reasoning, these models promote the safety assurance of the traditional reactive mechanisms to the proactive and context-sensitive paradigms. The paper has determined four fundamental areas of research, such as data-driven simulation, predictive reasoning, adaptive control, and trust-aware cognition, that together comprise the existing taxonomy of GenAI-based safety methods. The results given here demonstrate the current shift in the discipline to intelligent safety systems that can perceive, anticipate, and convey the dangers in common human–robot spaces.

In the review, some chronic limitations that limit the wider use of generative safety frameworks were also identified. They are scanty multimodal safety data, inadequate explainability in generative reasoning, computational burdens that do not allow real-time operation, and lack of standard verbal verification methodology. Additionally, the moral dilemmas, including the hallucination, prejudice, and transparency of the LLM-based reasoning, prove the criticality of the human supervision and responsible governance of AI. To resolve them, a cross-disciplinary approach is necessary involving the application of the knowledge of robotics, cognitive science, and AI ethics to make sure that generative intelligence is interpretable, certifiable, and trustworthy.

From an industrial perspective, the GenAI-driven safety frameworks reviewed in this paper offer measurable benefits across manufacturing, healthcare, and logistics sectors—collectively representing over 95% of the studied applications (Section 4.2). These benefits include reduced collision rates, faster reaction times to human motion, lower impact forces meeting ISO/TS 15066 requirements, and improved accuracy in safety instruction interpretation, as quantified in Section 4.5 and Section 7.5. However, industrial adoption also requires standardized benchmarking protocols, certification frameworks for learning-enabled systems, and open multimodal datasets—challenges identified throughout Section 6.1 and Section 6.4. Without these infrastructure elements, even technically superior systems will remain laboratory demonstrations rather than certified industrial solutions. It will be important that academia, industry, and regulatory bodies work together to create open and standardized benchmarks and create guidelines to test generative safety and certify it. In the future, the area is set to experience a change towards a mode of certifiable hybrid structures, a combination of deterministic safety logic and generative foresight. To achieve transparent and auditable safety systems, further progress in model interpretability, multimodal benchmarking, and ethical governance will be required. GenAI, being a cornerstone technology in HRC, will succeed solely because we can ensure a balanced approach to creativity and control so that generative systems do not just do things but also maintain human trust, responsibility, and wellbeing. Such synthesis provides a methodological and conceptual basis towards such an end, allowing the robotics community to proceed to the next stage of safe, adaptive, and ethically responsible human–robot interactions.

## Figures and Tables

**Table 1:** Comparison of this review with prior related surveys.

Aspect	This Review	Ajoudani [1]	Villani [3]	Gupta [35]	Giallanza [37]	Li [38]	Wang [36]
PRISMA methodology	✓	✗	✗	✗	✗	✗	✗
Generative AI focus	✓	✗	✗	Partial	✗	✗	Partial
HRC safety taxonomy	✓	✗	Partial	✗	✓	Partial	✗
ISO standard mapping	✓	✗	Partial	✗	Partial	✓	✗
Quantitative synthesis	✓	✗	✗	Partial	✗	✗	✗
Cognitive/ethical safety	✓	✗	✗	Partial	✗	✗	Partial
Certification roadmap	✓	✗	✗	✗	✗	✗	✗
Empirical vs. conceptual distinction	✓	✗	✗	✗	✗	✗	✗

**Table 2:** Inclusion and exclusion criteria.

Criteria Type	Description
Inclusion Criteria	1. Peer-reviewed journal or full conference paper 2. Written in English 3. Focus on safety in human–robot collaboration (HRC) 4. Integration of generative AI techniques (e.g., GANs, VAEs, LLMs) 5. Describes methodology or architecture for safety enhancement6. Includes evaluation, case study, or framework design
Exclusion Criteria	1. Not focused on HRC or safety 2. No generative AI component 3. Abstracts, posters, preprints, editorials 4. Duplicates or secondary reports 5. Not in English 6. Lacking full text or technical depth 7. Theoretical work without implementation or analysis

**Table 3:** Quality assessment checklist.

Quality Criterion	Assessment Question	Score Type
Q1	Are research goals clearly stated?	Yes/No
Q2	Is the safety framework or method well described?	Yes/No
Q3	Does the study use a generative AI model meaningfully?	Yes/No
Q4	Is the human–robot interaction context described clearly?	Yes/No
Q5	Is the evaluation method or results reliable?	Yes/No
Q6	Are limitations or assumptions discussed?	Yes/No

**Table 4:** Classification of reviewed studies by generative technique and domain.

GenAI Technique	Typical HRC Safety Applications	Representative Example(s)	Safety Contribution
GAN-based Simulation	Industrial cobots, assembly line stress-testing	Iklima et al. [28]—GAN + PSO generates self-collision-free robot trajectories	Synthetic hazard data for training; improved motion safety
VAE-based Motion Modeling	Human intent recognition, cooperative assembly	Ajoudani et al. [1]; Zhang et al. [36]	Probabilistic modeling of human motion for risk anticipation
Diffusion/Transformer Models	Predictive trajectory generation, dynamic workspace safety	Tian et al. [16]—TransFusion model for human-motion forecasting	Accurate motion prediction; proactive safety response
Generative Reinforcement/Adaptive Control	Real-time safety-constrained robot planning	Jabbour et al. [42]	Integration of generative priors in control for adaptive safe maneuvers
LLM-based Safety Frameworks	Service/assistive robots, human-in-the-loop safety decision-making	Kranz et al. [33]; Qi et al. [43]	Natural language hazard reasoning; improved transparency and trust

**Table 5:** Quantitative safety improvements reported for GenAI techniques in HRC.

GenAI Technique	Safety Metric	Improvement Reported	Comparison Baseline	Representative Studies	ISO Standard Relevance
GAN-based Simulation	Collision detection accuracy	+15–22%	Training without synthetic data	[49,50,51]	ISO/TS 15066 (detection)
VAE-based Motion Modeling	Human intent prediction error	28% reduction in position error	LSTM-based predictors	[1,36]	ISO/TS 15066 (separation monitoring)
Diffusion Models	Trajectory prediction accuracy	20–30% lower displacement error	Traditional recurrent networks	[34,52]	ISO/TS 15066 (speed monitoring)
Diffusion Models	Near-miss events	32% reduction	Baseline MPC controller	[36]	ISO 10218 (collision avoidance)
Transformer-based	Reaction time to sudden human motion	250 ms faster response	Classic safety-stop system	[38]	ISO 13849 (response time)
LLM-based Reasoning	User trust (7-point Likert scale)	+1.8 points	No explanation module	[37]	ISO/TR 14121-2 (psychological safety)
LLM-based Reasoning	Safety instruction interpretation	92% correct interpretation	Rule-based parsing	[35,38]	ISO/TS 15066 (human–robot communication)
Hybrid (GenAI + CBF)	Impact force during contact	40% reduction	Pure CBF controller	[36]	ISO/TS 15066 (power/force limiting)

**Table 6:** Experimental setup characteristics of representative generative AI-driven safety frameworks in human–robot collaboration.

Ref.	Sample Size	Experimental Setup Description	Data Type	Robot Platform	Control Law	Interaction Level
[36]	N = 18	Human–robot collaborative assembly in a shared workspace with participants performing sequential assembly tasks whereas robot predicts and avoids collisions.	Joint angles, point cloud	UR5 cobot	CBF-MPC	Close collaboration
[37]	Sim only	Simulated human–robot coexistence task in a virtual environment designed to test safe policy learning without physical risk.	Simulated states	KUKA LBR iiwa (simulated)	Lyapunov adaptive	Coexistence
[38]	N = 12	Mobile robot navigation in a crowded simulated corridor with fast-moving pedestrians to test socially compliant navigation.	Laser scan, RGB-D	Mobile robot (simulated/physical)	DWA + RL	Dynamic coexistence
[35]	500 real + synthetic	Hazardous event detection for collaborative assembly using both real human demonstrations and GAN-generated synthetic near-miss scenarios.	RGB-D, pose keypoints	UR10 assembly	Velocity scaling	Cooperative assembly
[34]	Human3.6M dataset	Trajectory forecasting from 3D skeleton data (perception-only study, no physical robot implementation).	3D skeleton sequences	N/A (perception only)	N/A	N/A
[39]	50 instructions	Natural language safety instruction interpretation in a simulated Fetch robot environment.	Natural language commands	Fetch manipulator (simulated)	Language-conditioned	Cooperation

**Table 7:** Methodological pipeline details for representative generative AI-driven safety frameworks.

Ref.	GenAI Model	Primary Safety Function	Processing Pipeline/Feature Extraction	Metric Definition and Formula (if Applicable)	Key Outcome
[36]	Diffusion + CBF	Collision avoidance	Pipeline: 1. 3D point cloud data from wrist-mounted cameras is preprocessed and normalized. 2. Diffusion model (denoising diffusion probabilistic model) predicts future human joint angles over a 2 s horizon with 10 Hz update rate. 3. Predictions are converted into time-varying safety constraints (minimum separation distance). 4. CBF-MPC controller solves an optimization problem subject to these constraints to generate safe robot trajectories. Feature extraction: Raw point clouds → skeletal joint angles → predicted trajectories.	Near-miss events: Instances where minimum distance $d_{min}$ < $d_{safe}$ (with $d_{safe}$ = 0.5 m) during task execution. Reduction percentage calculated as: ($N_{baseline}$ − $N_{ours}$)/$N_{baseline}$ × 100%, where $N_{baseline}$ is near-miss count with baseline MPC controller.	Near-miss −32%
[37]	Generative RL	Safe policy learning	Pipeline: 1. Simulated robot and human states (joint positions, velocities) are encoded as feature vectors. 2. Generative model samples multiple candidate actions from the current policy distribution. 3. Each candidate action is evaluated by a Lyapunov function to verify stability and safety before execution. 4. Only actions passing the verification are executed, and the policy is updated via reinforcement learning. Feature extraction: State vectors (dimension 24) encode robot configuration, human pose, and relative distance.	Trust: User-reported score on a 7-point Likert scale (1 = no trust, 7 = complete trust) collected via post-experiment questionnaire. Mean trust score calculated as (Σ scores)/$N_{participants}$. Improvement reported as difference between experimental (with explanation module) and control (without explanation) conditions.	Trust +1.8/7
[38]	Transformer + RL	Social navigation	Pipeline: 1. Laser scan data (720 points per scan) and RGB-D images (640 × 480) are fused. 2. Transformer encoder processes spatio-temporal dynamics of crowd movements, using self-attention to model interactions between all agents. 3. Encoded representation is fed to an RL policy (PPO) that generates velocity commands. 4. Commands are filtered by a safety layer that enforces minimum distance constraints. Feature extraction: Raw sensor data → agent positions and velocities → interaction features via attention weights.	Reaction time: Latency (ms) measured between a sudden human motion entering the robot’s safety zone and the robot’s first deceleration command. Calculated as $t_{response}$ = $t_{command}$ − $t_{intrusion}$, where $t_{intrusion}$ is timestamp when human enters predefined danger radius (1.5 m), and $t_{command}$ is timestamp when robot velocity drops below 50% of current speed.	React time −250 ms
[35]	GAN	Hazard augmentation	Pipeline: 1. Real human pose sequences from 10 participants performing assembly tasks are collected. 2. GAN generator creates synthetic pose sequences of near-miss events (sudden movements, unexpected reaches). 3. Synthetic data is mixed with real data at ratios of 25%, 50%, and 75% to create augmented training sets. 4. A CNN-based hazard detector (ResNet-50 architecture) is trained on augmented datasets. 5. Detector outputs hazard probability for each frame. Feature extraction: RGB-D images → pose keypoints (17 keypoints via OpenPose) → normalized keypoint coordinates.	Detection accuracy: Accuracy = (TP + TN)/(TP + TN + FP + FN), where TP = correctly identified hazardous events, TN = correctly identified safe events, FP = false alarms, FN = missed hazards. Improvement range (15–22%) represents best performance across different synthetic data mixing ratios.	Detection +15–22%
[34]	Diffusion (TransFusion)	Trajectory forecast	Pipeline: 1. 3D skeleton sequences (25 joints per frame) from Human3.6M dataset are normalized to a canonical coordinate system. 2. Transformer encoder processes past motion (2 s, 50 frames) to extract temporal features. 3. Diffusion model (TransFusion architecture) iteratively denoises random noise over 1000 steps to predict future 3D joint positions (1 s, 25 frames). 4. Classifier-free guidance adjusts prediction diversity vs. accuracy. Feature extraction: 3D joint coordinates → normalized joint positions (zero-centered, scaled) → temporal embeddings via transformer.	Displacement error: Mean Euclidean distance (mm) between predicted and ground truth 3D joint positions across the prediction horizon. Calculated as: Error = ($\frac{1}{J}$ × $\frac{1}{T}$) ∑J∑T ||$P_{pred}$(j,t) − $P_{gt}$ (j,t)||_2_, where J = number of joints (25), T = prediction frames (25), $P_{pred}$ and $P_{gt}$ are predicted and ground truth 3D positions. Improvement (20–30%) relative to LSTM baseline.	Displacement −20–30%
[39]	LLM (GPT-based)	Safety instruction interpretation	Pipeline: 1. Natural language safety instruction (e.g., “move slowly when I reach for the red box”) is tokenized using GPT tokenizer. 2. Pre-trained GPT model (GPT-3.5-turbo) generates a symbolic plan in PDDL (Planning Domain Definition Language) format. 3. Plan is parsed by a separate module into executable robot commands (navigation goals, speed constraints). 4. Commands are executed by the Fetch robot in simulation, with human operator verifying correctness. Feature extraction: Raw text → tokens → attention-weighted embeddings → PDDL symbols.	Instruction interpretation accuracy: Percentage of instructions where the robot’s final executed action matched the human’s intended task. Calculated as: Accuracy = $\frac{N_{correct}}{N_{Totol}}$ × 100%, where $N_{correct}$ = instructions correctly interpreted (verified by two independent human judges), $N_{Total}$ = 50 instructions.	92% correct interpret.

**Table 8:** Taxonomy summary of GenAI safety frameworks.

Framework Category	Primary Models	Target Safety Domain	Representative Studies	Key Outcomes
Data-Driven Simulation	GAN, VAE	Physical	[39,51,52]	Expanded safety-training data; improved robustness
Predictive Reasoning	Diffusion, Transformer	Physical/Cognitive	[40,41]	Accurate human-motion forecasting; proactive avoidance
Adaptive Control	Generative RL, Diffusion Control	Physical	[42]	Real-time adaptive policies; constraint satisfaction
Trust-Aware Cognition	LLM, Multimodal GenAI	Cognitive/Ethical	[43]	Transparent intent explanation; increased user trust

**Table 9:** Summary of identified challenges and future directions.

Challenge Domain	Current Limitations	Future Research Direction	Representative References
Data & Benchmarking	Limited shared datasets; poor multimodal diversity	Creation of standardized open HRC safety datasets	[44,45,51]
Model Interpretability	Black-box generative models hinder certification	Integration of XAI and symbolic reasoning layers	[46,47]
Real-Time Performance	High latency and memory cost in diffusion/transformer models	Lightweight, quantized architectures for edge robots	[39,59]
Validation & Certification	Lack of standardized verification and runtime assurance	Unified verification aligned with ISO safety standards	[41,48]
Ethical & Cognitive Safety	Hallucination and inconsistent reasoning in LLMs	Ethical alignment and human-in-the-loop evaluation	[43,49,50]

**Table 10:** Key insights of review.

Theme	Key Insight	Implication for Practice	Representative References
Hybrid Safety Integration	Generative models complement deterministic logic	Develop ISO-aligned hybrid architectures	[47,48,58,59,60,62]
Cognitive–Physical Convergence	Human trust requires transparent reasoning	Embed cognitive interpretability in HRC control	[45,49,50,63,64,65]
Benchmarking & Standards	Lack of reproducible evaluation	Build open HRC safety datasets and standards	[44,55,66,67,68,69]
Ethical & Governance Aspects	LLM-driven reasoning introduces bias and uncertainty	Create governance and audit frameworks	[43,49,56,70,71,72,73,74,75,76]
Industrial Impact	GenAI safety improves adaptability and trust	Validate through domain-specific case studies	[40,57,79,80,81]

## Data Availability

No new data were created or analyzed in this study.

## Supplementary Materials

The following supporting information can be downloaded at: https://www.mdpi.com/article/10.3390/s26072079/s1, Table S1: Complete PRISMA 2020 checklist with corresponding manuscript sections. Table S2: Detailed search queries and database-specific search strategies. Table S3: Quality assessment scores of all included studies based on the defined evaluation criteria. Table S4: Complete list of the 103 studies included in the systematic review, provided as a CSV/Excel file with metadata (authors, year, venue, generative AI method, safety domain, and key contributions).

## Abbreviations

The following abbreviations are used in this manuscript:

GenAI	Generative Artificial Intelligence
HRC	Human–Robot Collaboration
HRI	Human Robot Interaction
Cobot	Collaborative Robot
LLM	Large Language Models
PRISMA	Preferred Reporting Items for Systematic Reviews and Meta-Analyses
ISO/TS	International Organization for Standardization/Technical Specification
IEC	International Electrotechnical Commission
XAI	Explainable AI

## Appendix A. Search Strategy and Inclusion Criteria

This appendix details how studies were identified, screened, and included in accordance with the PRISMA 2020 framework.

The systematic search was conducted across IEEE Xplore, Scopus, ACM Digital Library, and SpringerLink databases between January 2015 and June 2025.

The following Boolean string was used with minor variations per database:

Search Query:

(“human–robot collaboration” OR “cobots” OR “collaborative robots”)

AND (“generative AI” OR “GAN” OR “diffusion model” OR “transformer” OR “LLM”)

AND (“safety” OR “hazard prediction” OR “trust” OR “risk assessment”).

Inclusion criteria:

1. Peer-reviewed studies focusing on generative or learning-based safety mechanisms in HRC.
2. Works employing simulation, reinforcement learning, diffusion, or LLMs for safety prediction, control, or reasoning.
3. Publications in English between 2015 and 2025.

Exclusion criteria:

1. Purely discriminative AI models without generative elements.
2. Studies without experimental, simulation, or safety evaluation results.
3. Non-peer-reviewed, review-only, or editorial materials.

After initial retrieval of 1134 records, duplicate removal and screening produced 850 unique studies. Following full-text assessment, 103 papers met the final inclusion criteria (78 qualitative + 25 quantitative), as visualized in Figure 2 (PRISMA diagram).

## Appendix B. List of Included Studies

The 103 studies included in the PRISMA synthesis are available in supplementary digital format (Excel/CSV) containing metadata (title, author, year, DOI, primary contribution, framework type).

Representative examples include studies addressing:

- Data-driven simulation: [44,45,51,58,60];
- Predictive reasoning: [38,47,59,63,64];
- Adaptive control: [39,42,53,77,79];
- Trust-aware cognition: [43,49,50,54,65].

The complete reference dataset can be made available upon request to ensure PRISMA transparency and reproducibility.

## Appendix C. PRISMA 2020 Compliance Summary

The review was structured and reported following the PRISMA 2020 Statement [78]. The complete checklist with section mapping is provided as Table S1 (Supplementary Materials).

Table A1 provides a condensed mapping of PRISMA checklist items to manuscript sections.

**Table A1:** PRISMA 2020 compliance S=summary.

Checklist Item	Description	Section Covered
Identification	Databases and queries described	Appendix A, Section 3
Screening	Inclusion/exclusion and duplicates	Section 3
Eligibility	Criteria applied for final inclusion	Section 3
Synthesis	Qualitative and quantitative synthesis	Section 5
Bias and Limitations	Discussed as methodological challenges	Section 6
Presentation	PRISMA diagram and taxonomy summary	Figure 3 and Figure 5
Reporting	Adheres to IEEE structure and ethical review	Section 1, Section 2, Section 3, Section 4, Section 5, Section 6, Section 7 and Section 8

## References

[1] Ajoudani A., Zanchettin A.M., Ivaldi S., Albu-Schäffer A., Kosuge K., Khatib O. (2018). Progress and prospects of the human–robot collaboration. Auton. Robot..

[2] Murashov V., Hearl F., Howard J. (2016). Working safely with robot workers: Recommendations for the new workplace. J. Occup. Environ. Hyg..

[3] Villani V., Pini F., Leali F., Secchi C. (2018). Survey on human–robot collaboration in industrial settings: Safety, intuitive interfaces and applications. Mechatronics.

[4] Wei Y., Xu Q. (2022). Design of a new passive end-effector based on constant-force mechanism for robotic polishing. Robot. Comput.-Integr. Manuf..

[5] Rinaldi G., Suglia V., Tiseni L., Camardella C., Xiloyannis M., Masia L., Buongiorno D., Bevilacqua V., Frisoli A., Chiaradia D. (2026). Towards a healthier workplace: How Flexos, an active and bilateral shoulder exoskeleton, provides support in weight-lifting and carrying tasks. IEEE Trans. Robot..

[6] Suglia V., Camardella C., Rinaldi G., Chiaradia D., Buongiorno D., Zhou H., Frisoli A., Leonardis D., Bevilacqua V. (2025). Muscle Network Analysis of a Dynamic Bilateral Task with an Upper Limb Exoskeleton. Proceedings of the 2025 International Conference on Rehabilitation Robotics (ICORR), Chicago, IL, USA, 2025.

[7] Suglia V., Camardella C., Rinaldi G., Chiaradia D., Buongiorno D., Leonardis D., Zhou H., Frisoli A., Bevilacqua V. (2026). Muscle networks analysis on an active occupational shoulder exoskeleton. Biomed. Signal Process. Control..

[8] Yang L., Zhang Z., Song Y., Hong S., Xu R., Zhao Y., Zhang W., Cui B., Yang M.-H. (2024). Diffusion models: A comprehensive survey of methods and applications. ACM Comput. Surv..

[9] Urain J., Mandlekar A., Du Y., Shafiullah M., Xu D., Fragkiadaki K., Chalvatzaki G., Peters J. (2024). Deep generative models in robotics: A survey on learning from multimodal demonstrations. arXiv.

[10] Goodfellow I., Pouget-Abadie J., Mirza M., Xu B., Warde-Farley D., Ozair S., Courville A., Bengio Y. (2020). GenerativeAdversarial Networks. Commun. ACM.

[11] Diederik P.K., Max W. (2019). An Introduction to Variational Autoencoders. Found. Trends Mach. Learn..

[12] Lynch C., Wahid A., Tompson J., Ding T., Betker J., Baruch R., Armstrong T., Florence P. (2022). Interactive language: Talking to robots in real time. arXiv.

[13] Croitoru F., Hondru V., Ionescu R.T., Shah M. (2023). Diffusion Models in Vision: A Survey. IEEE Trans. Pattern Anal. Mach. Intell..

[14] Koppula H.S., Saxena A. (2016). Anticipating human activities using object affordances for reactive robotic response. IEEE Trans. Pattern Anal. Mach. Intell..

[15] Laplaza J., Salazar E., Calinon S. (2024). Enhancing robotic collaborative tasks through contextual human motion prediction and intention inference. Int. J. Soc. Robot..

[16] Tian S., Zheng M., Liang X. (2023). TransFusion: A practical and effective transformer-based diffusion model for 3D human motion prediction. arXiv.

[17] Li W., Wang X., Zhang J., Orsag L. (2024). Safe human–robot collaboration for industrial settings: A survey. Int. J. Adv. Manuf. Technol..

[18] Vaswani A., Shazeer N., Parmar N., Uszkoreit J., Jones L., Gomez A.N., Kaiser Ł., Polosukhin I. (2017). Attention Is All You Need. Proceedings of the 31st International Conference on Neural Information Processing Systems (NIPS 2017), Long Beach, CA, USA, 4–9 December 2017.

[19] Huang X., Ruan W., Huang W., Jin G., Dong Y., Wu C., Bensalem S., Mu R., Qi Y., Zhao X. (2024). A survey of safety and trustworthiness of large language models through the lens of verification and validation. Artif. Intell. Rev..

[20] Pearl J. (2009). Causality: Models, Reasoning, and Inference.

[21] Huang W., Zhang X., Li Q., Sun Z., Li H. (2024). Formal verification of robustness and resilience of learning-enabled systems. Neurocomputing.

[22] Cai Z., Du X., Huang T., Lv T., Cai Z., Gong G. (2024). Robotic edge intelligence for energy-efficient human–robot collaboration. Sustainability.

[23] Wang S., Zhang J., Wang P., Law J., Calinescu R., Mihaylova L. (2024). A deep learning-enhanced digital twin framework for improving safety and reliability in human–robot collaborative manufacturing. Robot. Comput.-Integr. Manuf..

[24] Angleraud A., Ekrekli A., Samarawickrama K., Sharma G., Pieters R. (2024). Sensor-based human–robot collaboration for industrial tasks. Robot. Comput.-Integr. Manuf..

[25] Liu S., Zhao H. (2024). A survey on large language models for robotics. arXiv.

[26] Garcia-Gasulla D., Kestor G., Parisi E., Alberti-Binimelis M., Gutierrez C., Ghorab R.M., Montenegro O., Homs B., Moreto M. (2025). TuRTLe: A unified evaluation of LLMs for RTL generation. Proceedings of the 2025 ACM/IEEE 7th Symposium on Machine Learning for CAD (MLCAD), Santa Cruz, CA, USA, 8–10 September 2025.

[27] Yi S., Liu S., Yang Y., Yan S., Guo D., Wang X.V., Wang L. (2024). Safety-aware human-centric collaborative assembly. Adv. Eng. Inform..

[28] Iklima Z., Adriansyah A., Hitimana S. (2021). Self-collision avoidance of arm robot using generative adversarial network and particle swarm optimization (GAN-PSO). Sinergi.

[29] (2016). Robots and Robotic Devices—Collaborative Robots. https://www.iso.org/standard/62996.html.

[30] Suglia V., Palazzo L., Bevilacqua V., Passantino A., Pagano G., D’Addio G. (2024). A Novel Framework Based on Deep Learning Architecture for Continuous Human Activity Recognition with Inertial Sensors. Sensors.

[31] Yousif I., Samaha J., Ryu J., Harik R. (2024). Safety 4.0: Harnessing computer vision for advanced industrial protection. Manuf. Lett..

[32] Holland J., Kingston L., McCarthy C., Armstrong E., O’Dwyer P., Merz F., McConnell M. (2021). Service robots in the healthcare sector. Robotics.

[33] Kranz P., Schirmer F., Kaupp T., Daun M. (2024). Generative AI copilot to support safety analyses of human–robot collaborations: Hazard operability analysis and GPT-4. IEEE Softw..

[34] Tevet G., Gordon S.M., Hertz O., Haim H., Dubnov S., Kimmel R. (2022). Human motion diffusion model. arXiv.

[35] Gupta P., Ding B., Guan C., Ding D. (2024). Generative AI: A systematic review using topic modelling techniques. Data Inf. Manag..

[36] Wang T., Zheng P., Li S., Wang L. (2024). Multimodal human–robot interaction for human-centric smart manufacturing: A survey. Adv. Intell. Syst..

[37] Giallanza A., La Scalia G., Micale R., La Fata C.M. (2024). Occupational health and safety issues in human–robot collaboration: State of the art and open challenges. Saf. Sci..

[38] Li C., Zheng P., Zhou P., Yin Y., Lee C.K.M., Wang L. (2024). Unleashing mixed-reality capability in deep reinforcement learning-based robot motion generation towards safe human–robot collaboration. J. Manuf. Syst..

[39] Hu H., Ivanovic B., Liu C., Pavone M. (2024). Active uncertainty reduction for safe and efficient interaction planning: A shielding-aware dual control approach. *Int. J. Robot.* Res..

[40] Baumann D., Marco A., Turchetta M., Trimpe S. (2021). GoSafe: Globally optimal safe robot learning. Proceedings of the 2021 IEEE International Conference on Robotics and Automation (ICRA), Xi’an, China, 30 May–5 June 2021.

[41] Feng Z., Xue B., Wang C., Zhou F. (2024). Safe and socially compliant robot navigation in crowds with fast-moving pedestrians via deep reinforcement learning. Robotica.

[42] Jabbour J., Reddi V.J. (2024). Generative AI agents in autonomous machines: A safety perspective. Proceedings of the 43rd IEEE/ACM International Conference on Computer-Aided Design (ICCAD), New York, NY, USA, 27–31 October 2024.

[43] Qi Y., Huang W., Huang W., Zhao X., Mustafa M.A. (2024). Safety control of service robots with LLMs and embodied knowledge graphs. arXiv.

[44] Hagendorff T. (2024). Mapping the ethics of generative AI: A comprehensive scoping review. Minds Mach..

[45] D’Onofrio G., Sancarlo D. (2023). Assistive robots for healthcare and human–robot interaction. Sensors.

[46] Lee M., Liang X., Hu B., Onel G., Behdad S., Zheng M. (2024). A review of prospects and opportunities in disassembly with human–robot collaboration. J. Manuf. Sci. Eng..

[47] Althobaiti A., Hussain M.A., Aldossary M. (2024). How can LLMs and knowledge graphs contribute to robot safety? A few-shot learning approach. arXiv.

[48] Samsani S.S., Muhammad M.S. (2021). Socially compliant robot navigation in crowded environment by human behavior resemblance using deep reinforcement learning. IEEE Robot. Autom. Lett..

[49] Obrenovic B., Gu X., Wang G., Godinic D., Jakhongirov I. (2025). Generative AI and human–robot interaction: Implications and future agenda for business, society and ethics. AI Soc..

[50] Mökander J., Schuett J., Kirk H.R., Floridi L. (2024). Auditing large language models: A three-layered approach. AI Ethics.

[51] Kanazawa A., Kinugawa J., Kosuge K. (2019). Adaptive motion planning for a collaborative robot based on prediction uncertainty to enhance human safety and work efficiency. IEEE Trans. Robot..

[52] Gao J., Wang G., Xiao J., Zheng P., Pei E. (2024). Partially observable deep reinforcement learning for multi-agent strategy optimization of human–robot collaborative disassembly: A case of retired EV battery. Robot. Comput.-Integr. Manuf..

[53] Araujo H., Mousavi M.R., Varshosaz M. (2023). Testing, validation, and verification of robotic and autonomous systems: A systematic review. ACM Trans. Softw. Eng. Methodol..

[54] Winfield A.F.T., Michael K., Pitt J., Evers V. (2019). Machine ethics: The design and governance of ethical AI and autonomous systems. Proc. IEEE.

[55] Lara B., Ciria A., Escobar E., Gaona W., Hermosillo J. (2018). Cognitive Robotics: The New Challenges in Artificial Intelligence. Advanced Topics on Computer Vision, Control and Robotics in Mechatronics.

[56] Lettera G., Costa D., Callegari M. (2025). A hybrid architecture for safe human–robot industrial tasks. Appl. Sci..

[57] Tamantini C., Scotto di Luzio F., Hromei C.D., Cristofori L. (2023). Integrating physical and cognitive interaction capabilities in a robot-aided rehabilitation platform. IEEE Syst. J..

[58] Fisher M. (2021). An overview of verification and validation challenges for inspection robots. Robotics.

[59] Baumann D., Schön T.B. (2024). Safe reinforcement learning in uncertain contexts. IEEE Trans. Robot..

[60] Callari T.C., Vecellio Segate R., Hubbard E.-M., Daly A., Lohse N. (2024). An ethical framework for human–robot collaboration for the future people-centric manufacturing: A collaborative endeavour with European subject-matter experts in ethics. Technol. Soc..

[61] Yu Z., Zhang P., Shi J. (2026). Transformation of industrial robotics with natural language models: Recent progress and future prospects. Robot. Comput.-Integr. Manuf..

[62] Liu H., Wang L. (2017). Human motion prediction for human–robot collaboration. J. Manuf. Syst..

[63] Baidya S., Das S.K., Uddin M.H., Kosek C., Summers C. (2022). Digital twin in safety-critical robotics applications: Opportunities and challenges. Proceedings of the 2022 IEEE International Performance, Computing, and Communications Conference (IPCCC), Austin, TX, USA, 11–13 November 2022.

[64] Gu S., Kshirsagar A., Du Y., Chen G., Peters J., Knoll A. (2023). A human-centered safe robot reinforcement learning framework with interactive behaviors. Front. Neurorobot..

[65] Su H., Qi W., Chen J., Yang C., Sandoval J., Laribi M.A. (2023). Recent advancements in multimodal human–robot interaction. Front. Neurorobot..

[66] Taddeo M., Cowls J., Tsamados A., Taddeo M., Floridi L. (2024). Ethical approaches in designing autonomous and intelligent systems: A comprehensive survey towards responsible development. AI Soc..

[67] Yuan G., Liu X., Qiu X., Zheng P., Pham D.T. (2025). Human-robot collaborative disassembly in industry 5.0: A systematic literature review and future research agenda. J. Manuf. Syst..

[68] Zhong Y., Karthikeyan A., Pagilla P., Mehta R.K. (2024). Human-centric integrated safety and quality assurance in collaborative robotic manufacturing systems. CIRP Ann..

[69] Nascimento S., Prendergast C., Gallagher A., Holton D., Harron K. (2025). A deep dive into human-robot interaction in hospitals: Scoping review on the services provided, engagement behaviours and interaction outcomes. Int. J. Soc. Robot..

[70] Xiao J., Huang K. (2024). A comprehensive review on human–robot collaboration remanufacturing towards uncertain and dynamic disassembly. Manuf. Rev..

[71] Guerrier M., Ghignard F., Filliat D. (2024). Learning control barrier functions and their application in reinforcement learning: A survey. arXiv.

[72] Ragno L., Borboni A., Vannetti F., Amici C., Serrao M. (2023). Application of social robots in healthcare: Review on characteristics, requirements, and technical solutions. Sensors.

[73] Brunke L., Greeff M., Hall A.W., Yuan Z., Zhou S., Lorenz J., Schoellig A.P. (2022). Safe learning in robotics: From learning-based control to safe reinforcement learning. Annu. Rev. Control Robot. Auton. Syst..

[74] De Maio C., Di Gisi M., Fenza G., Gallo M., Loia V. (2025). A lifecycle-oriented survey of emerging threats and vulnerabilities in large language models. IEEE Access.

[75] Safavi F., Patel K., Vinjamuri R. (2025). Facial expression recognition with an efficient mix transformer for affective human-robot interaction. IEEE Trans. Affect. Comput..

[76] Lutin E., Elprama S.A., Cornelis J., Vanderborght B., Jacobs A. (2024). Pilot study on the relationship between acceptance of collaborative robots and stress. Int. J. Soc. Robot..

[77] Zeng Y., Yang Y., Zhou A., Tan J.Z., Tu Y., Mai Y., Klyman K., Pan M., Jia R., Song D. (2024). AIR-Bench 2024: A safety benchmark based on risk categories from regulations and policies. arXiv.

[78] Wang J., Lembono T.S., Kim S., Calinon S., Vijayakumar S., Tonneau S. (2022). Learning to guide online multi-contact receding horizon planning. Proceedings of the 2022 IEEE/RSJ International Conference on Intelligent Robots and Systems (IROS), Kyoto, Japan, 2022.

[79] Rehm M., Krummheuer A.L. (2024). When a notification at the right time is not enough: The reminding process for socially assistive robots in institutional care. Front. Robot. AI.

[80] Palazzo L., Suglia V., Grieco S., Buongiorno D., Brunetti A., Carnimeo L., Amitrano F., Coccia A., Pagano G., D’Addio G. (2025). A Deep Learning-Based Framework Oriented to Pathological Gait Recognition with Inertial Sensors. Sensors.

[81] Palazzo L., Suglia V., Grieco S., Buongiorno D., Pagano G., Bevilacqua V., D’ADdio G. (2025). Optimized Deep Learning-Based Pathological Gait Recognition Explored Through Network Analysis of Inertial Data. Proceedings of the 2025 IEEE Medical Measurements & Applications (MeMeA), Chania, Greece, 2025.

